# Perioperative Multimodal General Anesthesia Focusing on Specific CNS Targets in Patients Undergoing Cardiac Surgeries: The Pathfinder Feasibility Trial

**DOI:** 10.3389/fmed.2021.719512

**Published:** 2021-10-14

**Authors:** Akshay Shanker, John H. Abel, Shilpa Narayanan, Pooja Mathur, Erin Work, Gabriel Schamberg, Aidan Sharkey, Ruma Bose, Valluvan Rangasamy, Venkatachalam Senthilnathan, Emery N. Brown, Balachundhar Subramaniam

**Affiliations:** ^1^Department of Anesthesia, Critical Care, and Pain Medicine, Beth Israel Deaconess Medical Center, Harvard Medical School, Boston, MA, United States; ^2^Department of Anesthesiology, Lewis Katz School of Medicine at Temple University, Philadelphia, PA, United States; ^3^Department of Anesthesia, Critical Care and Pain Medicine, Massachusetts General Hospital, Boston, MA, United States; ^4^Picower Institute for Learning and Memory, Massachusetts Institute of Technology, Cambridge, MA, United States; ^5^Department of Anesthesia, Critical Care, and Pain Medicine, Riley Hospital for Children, Indiana University, Indianapolis, IN, United States; ^6^Department of Cardiothoracic Surgery, Beth Israel Deaconess Medical Center, Harvard Medical School, Boston, MA, United States; ^7^Department of Brain and Cognitive Sciences, Massachusetts Institute of Technology, Cambridge, MA, United States

**Keywords:** multimodal, analgesia, nociception, EEG, suppression, cardiac, regional, neuroanesthesia

## Abstract

Multimodal general anesthesia (MMGA) is a strategy that utilizes the well-known neuroanatomy and neurophysiology of nociception and arousal control in designing a rational and clinical practical paradigm to regulate the levels of unconsciousness and antinociception during general anesthesia while mitigating side effects of any individual anesthetic. We sought to test the feasibility of implementing MMGA for seniors undergoing cardiac surgery, a high-risk cohort for hemodynamic instability, delirium, and post-operative cognitive dysfunction. Twenty patients aged 60 or older undergoing on-pump coronary artery bypass graft (CABG) surgery or combined CABG/valve surgeries were enrolled in this non-randomized prospective observational feasibility trial, wherein we developed MMGA specifically for cardiac surgeries. Antinociception was achieved by a combination of intravenous remifentanil, ketamine, dexmedetomidine, and magnesium together with bupivacaine administered as a pecto-intercostal fascial block. Unconsciousness was achieved by using electroencephalogram (EEG)-guided administration of propofol along with the sedative effects of the antinociceptive agents. EEG-guided MMGA anesthesia was safe and feasible for cardiac surgeries, and exploratory analyses found hemodynamic stability and vasopressor usage comparable to a previously collected cohort. Intraoperative EEG suppression events and postoperative delirium were found to be rare. We report successful use of a total intravenous anesthesia (TIVA)-based MMGA strategy for cardiac surgery and establish safety and feasibility for studying MMGA in a full clinical trial.

**Clinical Trial Number:** www.clinicaltrials.gov; identifier NCT04016740 (https://clinicaltrials.gov/ct2/show/NCT04016740).

## Introduction

General anesthesia is a drug-induced reversible state consisting of unconsciousness, amnesia, antinociception, and immobility while maintaining physiological stability (1). The primary objective of general anesthesia is to eliminate pain during surgery and invasive diagnostic procedures. The state of general anesthesia eliminates pain by preventing both nociception (the transmission of noxious neural sensory signals) and conscious perception of nociception. Balanced anesthesia is defined by the administration of a combination of agents to achieve the anesthetic state. The current practice of balanced anesthesia typically uses opioids for antinociception, intravenous propofol to induce and inhaled ethers to sustain unconsciousness and produce amnesia, and muscle relaxers for immobility (1, 2). However, recent advances in understanding specific neural circuits involved in antinociceptive and arousal pathways allows for the synergistic use of medications to achieve anesthesia while reducing total anesthetic agent exposure (3). For example, antinociceptive medications such as ketamine and magnesium additionally contribute to unconsciousness by decreasing arousal (4). For cardiac surgeries, synergistic effects may be important in reducing the dosing requirement for anesthetics that affect hemodynamic stability such as propofol, or reducing consumption of opioids (5, 6).

Multimodal general anesthesia (MMGA) is a strategy for leveraging known central and peripheral neurophysiology to control levels of unconsciousness and antinociception during anesthetic practice. MMGA uses a combination of multiple agents with specific central nervous system targets at low doses to maximize antinociceptive and sedative effects while minimizing the potential side effects of each agent. Elderly patients undergoing cardiac surgery are at a high risk for adverse consequences in the perioperative period including comparatively higher rates of morbidity, mortality, complication rates, repeat hospital admissions, and healthcare utilization (7–9). Postoperative delirium and intraoperative hemodynamic instability are also more common in elderly cardiac surgical patients due to factors such as decreased cognitive function, limited cardiac and autonomic reserve, and increased susceptibility to deeper anesthetic states (10–12). More generally, cardiac anesthesiology remains challenging due to the high-risk nature of bypass and valve procedures, distinct mean arterial pressure (MAP) goals at different phases of surgery, and repeated noxious stimuli.

Interference of the surgical procedure with the typical signs used to titrate anesthetic agents further complicate cardiac anesthesiology. Blood pressure and heart rate no longer provide insight into nociception during cardiac bypass. Therefore, during cardiac MMGA, anesthetists may utilize electroencephalography (EEG) to appropriately titrate anesthetic dosing to suppress consciousness while avoiding deleterious neural states such as burst suppression. Burst suppression is a deep state of anesthetic-induced coma that has been associated with increased likelihood of perioperative neurocognitive disorders (PND)] (13–15). Previous research has suggested that EEG-guided anesthetic management does not prevent postoperative delirium after major surgery (16). However, this previous research utilized bispectral index (BIS)-based EEG guidance, which only reduced anesthetic dosing by 0.11 minimum alveolar concentration (MAC). Additionally, elderly patients compared to a non-elderly patient population demonstrate both increased burst suppression for the same anesthetic dose and changes in thalamocortical function reflected in reduced alpha-band power and coherence on the EEG waveform during general anesthesia (17, 18).

In the PATHFINDER (Perioperative multimodal general AnesTHesia FocusINg on specific CNS targets in patients undergoing carDiac surgERies) pilot study, we used an MMGA strategy for elderly patients (age ≥60 years) undergoing cardiac surgery with cardiopulmonary bypass. We achieved antinociception using a combination of intravenous remifentanil, ketamine, dexmedetomidine, magnesium, and bupivacaine given as a pecto-intercostal fascial (PIFB) regional block before the start of surgery. We maintained patient unconsciousness primarily using propofol. However, the chosen antinociceptive agents also decrease arousal and therefore contribute to maintenance of unconsciousness. Because we achieved amnesia via suppression of consciousness, we avoided the use of midazolam or other benzodiazepines due to known dangers regarding midazolam in older patients (19). Monitoring the level of unconsciousness was facilitated by the interpretation of unprocessed EEG waveforms in combination with the EEG spectrogram. We aimed to: (1) determine the feasibility of using EEG-guided MMGA anesthesia during cardiac surgeries, (2) perform exploratory analysis of hemodynamic stability and vasopressor usage in comparison to a previously collected cohort, and (3) perform exploratory analysis of intraoperative EEG suppression events and postoperative delirium.

## Materials and Methods

### Multimodal General Anesthesia: Rational Design of an Anesthesia Strategy From Neurophysiology

#### Antinociception

**Figure 1:**
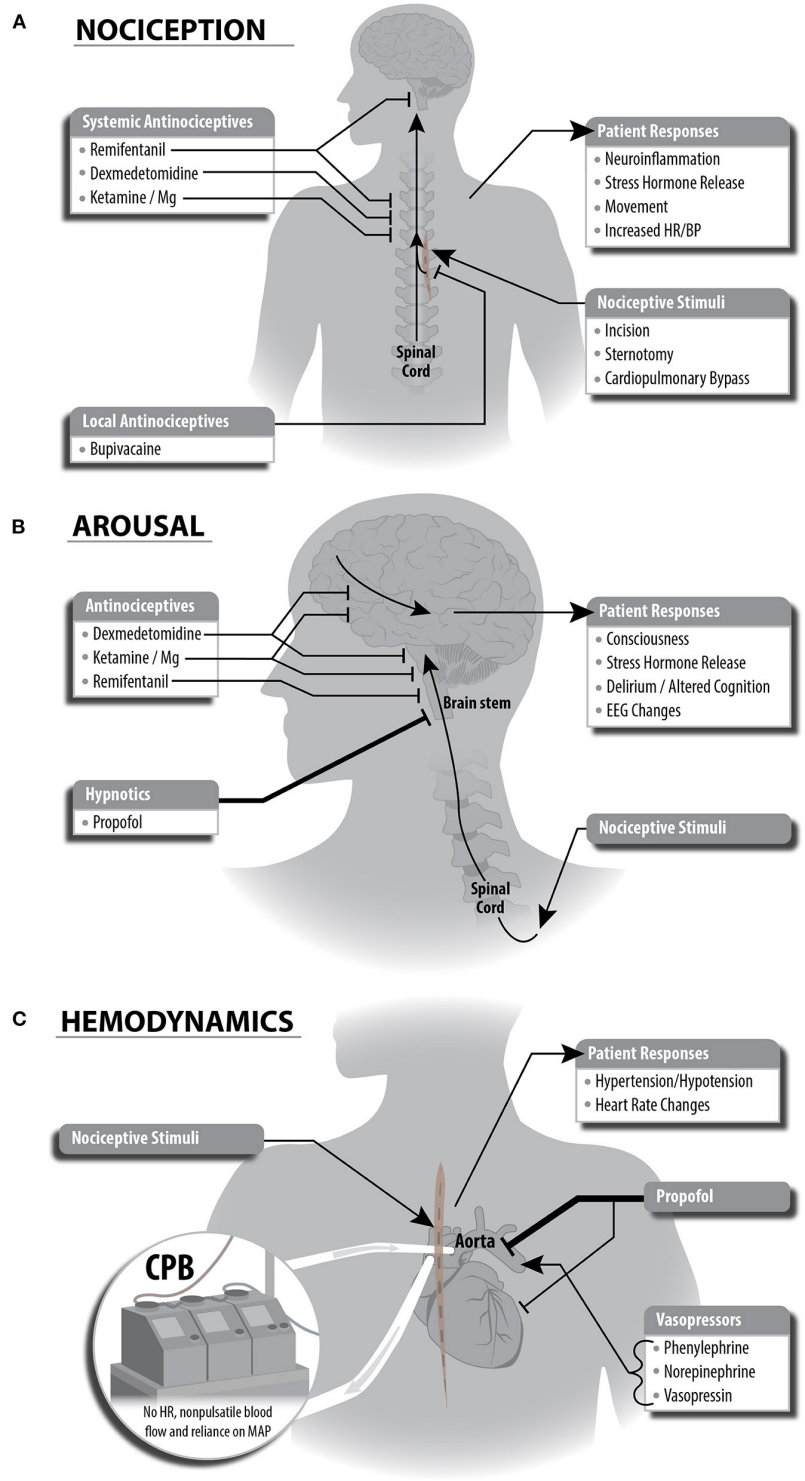
Schematic depictions of multimodal general anesthesia protocol split into individual components for anesthetic plan. **(A)** Nociceptive stimuli are managed with local and systemic anitinociceptives. This strategy leverages complementary mechanisms of each antinociceptive drug to achieve inhibition of nociceptive responses locally, at sites within the spinal cord, and at sites in the brain stem. **(B)** Decreased arousal (unconsciousness) is generated primarily via propofol potentiation of GABAergic inhibitory neurotransmission. The antinociceptive agents we selected have secondary effects in cortical or brain stem regions and contribute to decreased arousal. **(C)** Cardiopulmonary bypass (CPB) and anesthetic agents affect hemodynamic stability during cardiac surgery. Administration of propofol causes bradycardia and hypotension, and the anesthesiologist will guide administration of vasopressors to compensate for these changes. Importantly, CPB eliminates heart rate and pulsatile blood flow, causing the anesthesiologist to rely on mean arterial blood pressure to guide vasopressor dosing. This also eliminates the ability to use heart rate as a signal of patient response to painful stimuli.

We employed a combination of remifentanil, ketamine, magnesium, dexmedetomidine, and bupivacaine for antinociception. Remifentanil targets multiple classes of opioid receptors in both the PNS to block transmission of nociceptive signals to the spinal cord and in the CNS to increase inhibition of nociceptive signaling via descending pathways from periaqueductal gray. Ketamine and magnesium are thought to decrease nociception in the PNS by targeting N-methyl-D-aspartate receptor (NMDA) glutamate receptors in the dorsal horn of the spinal cord, thus decreasing ascending excitatory nociceptive input to the spinal cord and the CNS. Ketamine is an NMDA receptor antagonist, while magnesium plugs open NMDA channels. Dexmedetomidine potentiates inhibitory interneurons that synapse onto the dorsal horn, thus achieving synergy with ketamine and magnesium by potentiating descending antinociceptive neurotransmission. We also used magnesium as a membrane stabilizer at the end of cardiopulmonary bypass following cross clamp removal. Bupivacaine is a local antinociceptive agent with a less-established mechanism, however, it is a known sodium channel antagonist, thus causing localized inhibition of nociceptive signaling. Bupivacaine was given as a pecto-intercostal fascial block before the start of surgery. The primary hypnotic, propofol, contributes secondarily to antinociception, by removing awareness of noxious stimuli throughout the procedure. Suppressing nociceptive signals locally, at the spinal cord, and within the brain enables a more complete blockage of nociception (see Figure 1A).

#### Unconsciousness

We used a propofol infusion as the primary hypnotic agent to maintain unconsciousness. Propofol produces sedation and unconsciousness via potentiation of GABAergic inhibition within the cortex, thalamus, brainstem, and spinal cord (see Figure 1A). Propofol-induced unconsciousness causes a drug- and age-specific spectral pattern in frontal EEG demonstrated by a predominance of large amplitude slow (<1 Hz), delta (1–4 Hz), and alpha (8–12 Hz) oscillations. This EEG pattern shifts through a reduction in alpha power as the brain ages, although the extent of this change varies between individuals (20). These specific patterns enable titration of intravenous propofol throughout a surgical procedure when compared to volatile anesthetics such as sevoflurane, which exhibit increased electroencephalogram power across a range of frequencies except at slow oscillations and the propofol alpha oscillation peak (21). Therefore, we used a total intravenous anesthetic (TIVA) approach given recent evidence demonstrating non-inferiority to anesthesia with a volatile agent in regard to 1-year mortality in cardiac surgery (22).

Secondarily, antinociceptive agents assist in maintaining unconsciousness (see Figure 1B). Opioids decrease arousal through inhibition of cholinergic circuitry in the brainstem. Low doses of ketamine induce altered arousal and hallucination via its high specificity as an antagonist of NMDA receptors on GABAergic inhibitory cortical interneurons, thus disinhibiting cortical pyramidal cells. At higher doses, ketamine inhibits cortical pyramidal cells via NMDA receptor antagonism. Magnesium acts broadly across the CNS and likely enhances inhibition (and thus hypnosis) by also blocking NMDA receptors, though with less specificity. Dexmedetomidine reduces arousal via multiple mechanisms including (1) suppression of norepinephrine release from the locus coeruleus to the basal forebrain, thalamic intralaminar nucleus, hypothalamic preoptic area, and cortex and (2) disinhibition of GABAergic and galanergic inhibitory interneurons which project to arousal nuclei.

#### Hemodynamics

Maintenance of hemodynamic stability during cardiac surgery is a challenge due to both mechanical disruption of cardiac function and hemodynamic effects of anesthetic agents. Agents used in our MMGA strategy to maintain a state of unconsciousness impact cardiovascular function and blood pressure during cardiac surgery. Systolic hypotension and bradycardia are the most common hemodynamic side effects of propofol and dexmedetomidine (see Figure 1C). These effects are mediated by inhibition of sympathetic vasoconstriction by propofol, and agonism of α-2 receptors by dexmedetomidine (23, 24). In contrast, nociceptive stimuli often cause an increase in heart rate and blood pressure. To maintain hemodynamic stability, we used phenylephrine (sympathomimetic α-adrenergic receptor activator), norepinephrine (sympathomimetic with primarily α-adrenergic receptor activator), and vasopressin (non-apeptide with vascular smooth muscle receptor activator), vasopressors commonly used in cardiac surgery. During cardiopulmonary bypass, non-pulsatile blood flow and lack of heart rate readings generates a reliance on MAP for hemodynamic control.

#### Immobility

We used rocuronium, a nicotinic acetylcholine receptor antagonist, as the primary agent for sustaining immobility throughout the surgery. In addition, magnesium is a muscle relaxant through its role as an NMDA antagonist, and propofol inhibits spinal cord alpha motor neurons to contribute to immobility.

### Monitoring Unconsciousness and Antinociceptive State

We designed our MMGA strategy to enable use of the raw EEG waveforms, density spectral array, and processed EEG score to ensure individualized care and adequate levels of unconsciousness throughout the study protocol. Typically, anesthesiologists tightly regulate hemodynamics and alter them to accommodate the surgeon and specific surgical steps. They also monitor changes in cardiac output and hemodynamics to adjust hypnotic and antinociceptive dose. However, during cardiac surgery and especially during cardiac bypass, vital signs such as heart rate and blood pressure are less reliable due to non-pulsatile blood flow and surgical preference for particular hemodynamic goals. Thus, other methods such as direct brain monitoring may be used to personalize anesthetic care and monitor levels of unconsciousness and antinociception.

EEG-monitored anesthetic practice often relies on processed EEG scores, with numbers given from zero to one hundred to infer the depth of consciousness. Lower numbers refer to known potentially deleterious patterns on the raw EEG waveform, such as burst suppression. Rather than solely relying on an EEG processing algorithm such as BIS, in this study we trained clinicians to recognize raw EEG signals corresponding to unconsciousness and to adjust dosing to avoid EEG signals corresponding to arousal or coma-like states.

### Multimodal Anesthesia Protocol During Cardiac Surgery

Cardiac surgery involves repeated noxious stimuli, and therefore anesthetic agents must be titrated in varying doses to adequately suppress consciousness and nociception throughout a cardiac surgery. Here, we present our approach (see Table 1).

**Table 1:** Approach for MMGA used in this study.

**Surgical phase**	**Noxious stimulus**	**Equivalent MAC to suppress noxious stimulus**	**Standard**	**TIVA: Dexmedetomidine**	**TIVA: Propofol**	**TIVA: Ketamine**	**TIVA: Remifentanil**
Induction	⊕⊕	1.5–2.0	500 mcg Fentanyl; 50–100 mg Propofol	Start after intubation; 0.2 mcg · kg^−1^ · hr^−1^	50–100 mcg · kg^−1^ · hr^−1^	None	None
Line placement	⊖	0.6	Lower anesthetic	No change	Titrate to EEG; may be able to decrease	None	None
Incision	⊕ ⊕ ⊕	1.5 – 2.0	250 mcg Fentanyl	No change	Titrate to EEG	None	Start in anticipation of pain 5 minutes before; 0.1–0.2 mcg·kg^−1^·min^−1^
Median sternotomy	⊕ ⊕ ⊕	2	250 mcg Fentanyl	No change	Titrate to EEG	Start before sternotomy at 0.2 mg · kg^−1^ · hr^−1^	Increase in anticipation of pain; 0.2 mcg· kg^−1^·min^−1^
Internal mammary artery dissection	⊖	0.6	Lower anesthetic	No change	Titrate to EEG; may be able to decrease	No change	Lower the dose to 0.05 mcg·kg^−1^·min^−1^
Chest retractor	⊕	1	Increase isoflurane	No change	Titrate to EEG	No change	Increase the dose to 0.2 mcg·kg^−1^·min^−1^
Pericardial incision	⊕ ⊕ ⊕ ⊕ ⊕	1.3	High dose of inhalational agents	No change	Titrate to EEG	No change	Keep at 0.2 mcg·kg^−1^·min^−1^ or more
Aortic cannula	Neutral	As needed for MAP goals	MAP 90; Increase isoflurane to lower pressure	No change	Titrate to EEG; may be able to decrease	No change	Lower the dose as per MAP goals; SBP 90 mm of Hg
Venous Cannula	Neutral	As needed for MAP goals	SBP 100–120; lower anesthetic	No change	Titrate to EEG	No change	Lower the dose as per MAP goals; SBP 100–120 mm of Hg
Bypass-cold	⊖	Perfusionist administered to MAP 60 mm of Hg	34°C; Increased volume of distribution; Lower anesthetics	No change	Increase due to volume of distribution	No change	Increase due to volume of distribution
Bypass-rewarming	⊕ ⊕ ⊕	Perfusionist administered to MAP 60 mm of Hg	37°C; Dose of Midazolam (2 mg) to avoid awareness	No change	Increase	No change	Increase
Chest closure	⊖	Dose adjusted to keep the SBP close to 100 mm of Hg	Lower anesthetics; Chest closure will impede venous return and decrease BP	No change	Titrate to EEG	Stop after chest closure	Stop after chest closure; Consider 0.2–0.4 mg of hydromorphone bolus
Transport	⊖⊖	Stop	IV Propofol 20–40 mcg·kg^−1^·hr^−1^	0.4–1.4 mcg· kg^−1^ · hr^−1^	Stop	Stop	Stop

#### Induction and Intubation

Induction was performed with 250 mcg of IV fentanyl, 50–150 mg of propofol, or 10–20 mg of etomidate and endotracheal intubation was facilitated by 50–100 mg of IV rocuronium. No volatile anesthetic was used at any point during the case. After intubation, infusions of propofol at 50–100 mcg·kg^−1^ ·hr^−1^ and dexmedetomidine at 0.2 mcg·kg^−1^·hr^−1^ were started while the patient was being draped and prepped for surgical incision.

#### Central Line Placement

During the placement of the central line and for the remainder of the study procedure, dexmedetomidine and propofol were titrated to induce adequate levels of unconsciousness while avoiding burst suppression patterns by trained fellows and attending anesthesiologists.

#### Pecto-Intercostal Fascial Regional Block

**Figure 2:**
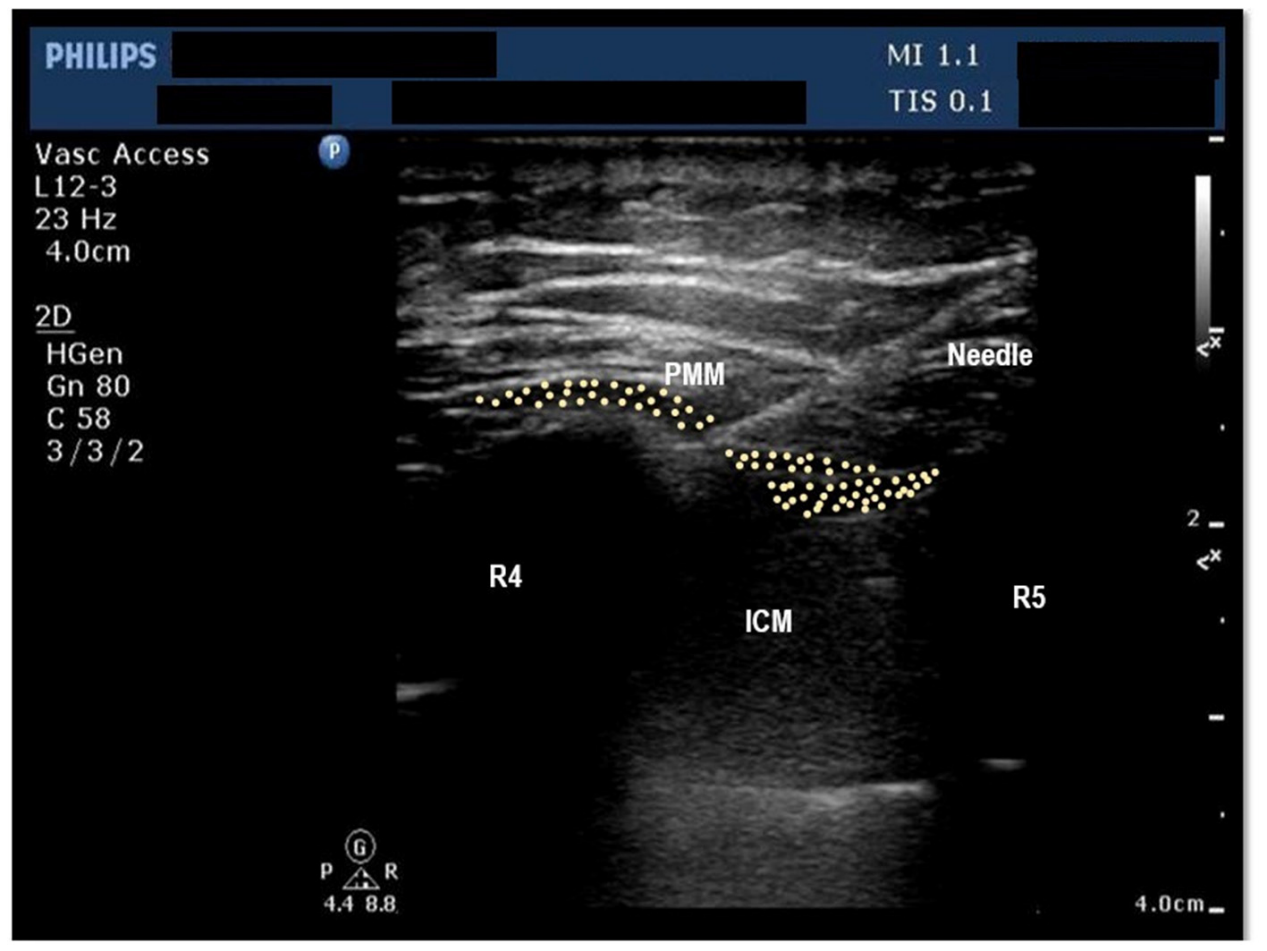
Ultrasound image of the chest wall showing anterior chest wall structures at the level of 4th Intercostal space. PMM, Pectoralis major muscle, ICM, Intercostal Muscles, R4, Fourth rib, R5, Fifth rib, Shows the position of needle, pleura and lung parenchyma.

After the placement of the central line, trained anesthesiologists administered a bilateral pecto-intercostal fascial block (PIFB) using 20 mL of 0.25% bupivacaine on both sides of the chest (total of 40 mL). Ultrasound guidance was used to administer the PIFB in the superficial plane between the external intercostal and the pectoralis major muscles (see Figure 2).

### Chest Incision, Sternotomy, Chest Retraction, and Pericardial Incision

An infusion of remifentanil was started at 0.1–0.2 mcg·kg^−1^·min^−1^ after central line placement and the PIFB. Approximately three to five min before chest incision, the anesthesiologist increased remifentanil to approximately 0.2 mcg·kg^−1^·min^−1^ in anticipation of noxious stimuli. During sternotomy, pericardial incision, and chest retraction, remifentanil was maintained at approximately 0.2 mcg·kg^−1^·min^−1^ (three to five min prior to the anticipated stimulus) because of the anticipated noxious stimuli and hemodynamic changes involved with these surgical procedures. If warranted by adequate levels of unconsciousness and hemodynamic goals, the anesthesiologist would attempt to decrease the rate of infusion of propofol due to the gradual loss of power within the alpha frequency range during surgery in elderly patients (20). It is important to note that all the infusions were backed by a constant micro-drip infusion of a crystalloid.

### Internal Mammary/Radial Artery Dissection, Aortic and Venous Cannula Placement

During the dissection of the internal mammary/radial artery, the anesthesiologist lowered remifentanil to approximately 0.05 mcg·kg^−1^·min^−1^ per MAP goals due to lower levels of noxious stimuli. The anesthesiologist adjusted the infusion of remifentanil during the placement of the aortic and venous cannula to maintain systolic blood pressure goals of roughly 90–120 mm of Hg.

#### Cardiopulmonary Bypass

Throughout cardiopulmonary bypass, anesthesiologists controlled the anesthetic regimen rather than perfusionists. Perfusionists were allowed to manage vasopressor agents during this time period. Despite attempts to minimize dosages of agents, some patients (especially those who had decreased intra-operative power within the alpha and beta range) still underwent electroencephalogram burst suppression (25), especially during our institution's standard protocol of giving 5 mg of magnesium as a membrane stabilizer following cross clamp removal. At the onset of cardiopulmonary bypass, however, an increased volume of distribution (due to cardiopulmonary bypass reservoir volume) often necessitated an increase in the propofol infusion by approximately 10–20 mcg·kg^−1^·hr^−1^ from previous levels to minimize levels of burst suppression.

#### Chest Closure and ICU Transfer

After chest closure, the anesthesiologist maintained dexmedetomidine at the baseline dosage (approximately 0.2 mcg·kg^−1^·hr^−1^) while stopping the propofol, ketamine, and remifentanil infusions.

Before transportation to the intensive care unit, the patient received a bolus of approximately 0.2–0.4 mg of intravenous hydromorphone. The anesthesiologist increased the rate of the dexmedetomidine infusion (0.4–1.4 mcg·kg^−1^·hr^−1^) from operating room transport until intensive care unit extubation based on patient response, hemodynamic stability, and adequate levels of consciousness on the electroencephalogram. The objective of the dexmedetomidine infusion was to maintain sedation and hemodynamic stability until the patient was settled in the intensive care unit. The rate of the dexmedetomidine infusion was increased in order to provide sedation in the absence of the propofol infusion; however, a propofol infusion was an option for addition or replacement of sedation until extubation based on the treating physician's discretion.

Acetaminophen was administered within 1 h of ICU admission and thereafter every 6 h for 4 doses. Both groups received opioids and other analgesics according to standard care practices during these 48 h. A trained staff member (BS or AS) was available for guidance in the ICU throughout the study procedures. Upon extubation, a study member stopped the electroencephalogram recording.

### Regulatory Measures

Registration of the PATHFINDER feasibility trial occurred prior to the start of the trial and any patient enrollment undertaken (ClinicalTrials.gov Identifier: NCT04016740; Principal Investigator B.S.; First Registered Date July 9, 2019). We conducted this non-randomized prospective observational feasibility trial on 20 patients at Beth-Israel Deaconess Medical Center in Boston, Massachusetts from July 2019 to January 2020 (IRB Number: 2019-P-000407; Principal Investigator B.S.; Date of Approval July 12, 2019). The Committee on Clinical Investigations granted institutional review board approval, and all patients provided written informed consent. This manuscript adheres to the applicable CONSORT guidelines.

### Sample Size

The PATHFINDER cohort recruited 22 patients in total in order to ascertain the feasibility of implementing this intervention in the cardiac operating rooms and cardiovascular intensive care units at the Beth-Israel Deaconess Medical Center. Two patients were assigned to a small control cohort used to test EEG recording and data collection in the OR, the remaining 20 patients were assigned to the MMGA cohort. Two of the 20 patients in the MMGA cohort were excluded from the analysis; one patient was enrolled but study staff were not available to perform the protocol on the day of surgery, and another patient did not have proper operative timepoints (e.g., sternotomy) recorded during their procedure. In total, 2 patients were included in the control cohort and 18 patients were included in the MMGA cohort (see Table 2).

**Table 2:** Demographics of study cohorts.

**Demographics**	**MMGA Cohort** **(*n* = 18)**	**MMGA Control** **(*n* = 2)**	**DEXACET Control** **(*n* = 59)**
**Sex, no (%)**			
Male	13 (72.2)	1 (50)	50 (84.7)
Female	5 (27.7)	1 (50)	9 (15.3)
Age, mean (SD)	72.7 (5.2)	67 (9.9)	70 (13.0)
Weight, mean (SD), kg	82.1 (17.6)	88.4 (0.3)	90 (24.4)
Height, mean (SD), cm	170.7 (9)	166.4 (1.8)	173 (11.4)
**ASA status, no (%)**			
ASA 3	9 (50)	1 (50)	
ASA 4	9 (50)	1 (50)	
Length of surgery, mean (SD), min	346.5 (100.4)	338.5 (123.7)	
**Type of surgery, no (%)**			
Isolated CABG	8 (44.4)	1 (50)	41 (69.5)
Isolated valve	4 (22.2)	1 (50)	
CABG + valve	5 (27.8)		14 (22.0)
Other	1 (5.6)		5 (8.5)

### Inclusion and Exclusion Criteria for PATHFINDER MMGA and Control Cohorts

Patients aged 60 years or older undergoing coronary artery bypass graft surgery with or without aortic and/or mitral valve replacement requiring cardiopulmonary bypass were eligible for trial inclusion. Patients with preoperative left ventricular ejection fraction of less than 30%, cognitive impairment as defined by total Montreal Cognitive Assessment (MoCA) score < 10, significant visual impairment, liver dysfunction, recent history of drug or alcohol misuse (defined as greater than 2 drinks per day), active (in the past year) history of alcohol abuse (defined as greater than or equal to 5 drinks/day for men or 4 drinks/day for women), chronic opioid use for chronic pain conditions with tolerance (defined as a total daily dose of an opioid at or more than 30 mg morphine equivalent for more than one month within the past year), English-language limitations, and hypersensitivity to study medications and patients undergoing emergent surgery were excluded.

### Historical Cohort for Hemodynamic Comparison

We used the control cohorts of data collected previously in the DEXACET clinical trial (non-acetaminophen groups, ClinicalTrials.gov Identifier: NCT02546765) to construct a historical cohort for comparison with the PATHFINDER MMGA hemodynamic stability and vasopressor doses (26). The study participants were selected similarly to the PATHFINDER cohort: The eligible population consisted of patients 60 years of age or older undergoing coronary artery bypass graft surgery with or without aortic and/or mitral valve replacement requiring cardiopulmonary bypass. We excluded patients with a preoperative left ventricular ejection fraction of less than 30%, preexisting cognitive impairment, Alzheimer's disease, Parkinson's disease, prescribed medications for treating cognitive decline, history of recent seizures, serum creatinine levels above 2 mg/dL, liver dysfunction, recent history of alcohol misuse, English-language limitations, and hypersensitivity to study medications and patients undergoing emergent surgery.

### EEG Data Recording and Postoperative Data Analyses

**Figure 3:**
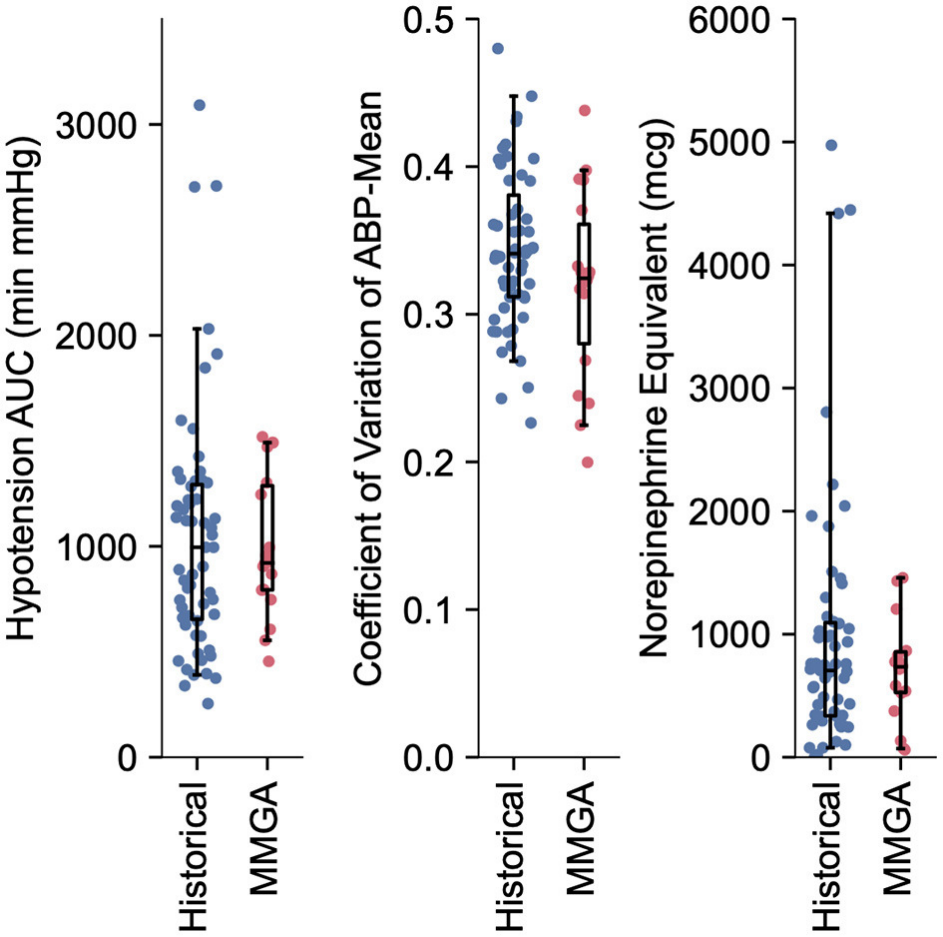
Quantification of hemodynamic stability and vasopressor usage during MMGA in comparison to a historical standard-of-care. Little difference was observed between hypotensive AUC (median, [IQR] min'mmHg; MMGA 921.0, [793.1, 1287.2]; historical 995.1, [653.7, 1292.7]; *P* = 0.72 Mann-Whitney U test), CV (median, [IQR]; MMGA 0.32, [0.28 0.36]; historical 0.34, [0.31, 0.38]; *P* = 0.14 Mann-Whitney U test), and norepinephrine equivalent dose (median, [IQR] mcg; MMGA 734.5, [526.1, 856.1]; historical 704.77, [336.4 1065.0], *P* = 0.90 Mann-Whitney U test).

EEG from the PATHFINDER control cohort (*n* = 2) and MMGA cohort (*n* = 18) was recorded intraoperatively and analyzed postoperatively for retrospective analysis. EEG data were recorded with a pre-amplifier bandwidth of 0.5 to 92 Hz, sampling rate of 178Hz, with 16-bit, 29 nV resolution. The standard Sedline (Masimo Corporation, Irvine, CA, United States) electrode array records from electrodes located approximately at positions Fp1, Fp2, F7, and F8, with ground electrode at Fpz, and reference electrode approximately 1 cm above Fpz. Electrode impedance was less than 5 kΩ in each channel. The EEG was analyzed using the multi-taper spectrogram intraoperatively to manage unconscious and nociceptive state, and postoperatively to retrospectively observe unconsciousness (see Figure 3). EEG was also analyzed for suppression events, which are common during coma-like deep states of unconsciousness. Suppression identification was achieved using a recursive variance tracking algorithm (27) with an adaptive threshold that was determined by a second estimate of the variance evolving on a slower timescale. Suppressions are thus characterized as segments of EEG where the local variance is small relative to the long-term baseline variance. All data analyses were performed using the scientific Python stack (28).

### Intraoperative Hemodynamic and Vasopressor Data Recording and Postoperative Data Analyses

Mean arterial blood pressure (ABP-mean) was recorded in 15-s intervals throughout the surgical case using the Anesthesia Information Management System (CompuRecord; Philips Healthcare, Andover, MA). Vasopressor boluses and infusions were recorded in electronic medical records. We calculated the area under the 65 mm Hg ABP-mean curve (AUC) using the trapezoidal rule. We calculated the coefficient of variation (CV) of ABP-mean to quantify intraoperative stability of blood pressure. We calculated the total vasopressor-inotrope dose in norepinephrine-equivalent (NE) mcg using the following formula:


$$\begin{array}{l} \text{Norepinephrine Equivalent mcg }=\;k_{1}N\;+\;k_{2}E\;+\;k_{3}P\\ \;+\;k_{4}M\;+\;k_{5}V \end{array}$$

Where N, E, P, M, and V are total doses of norepinephrine, epinephrine, phenylephrine, milrinone, and vasopressin, respectively. This equation was previously defined in terms of rates (29, 30) enabling us to derive conversion factors *k*_1−5_ for total dose as made explicit in Table 3. We compared AUC, CV, and vasopressor-inotrope total doses between the historical cohort (*n* = 59) and the PATHFINDER MMGA cohort (*n* = 18).

**Table 3:** Equation terms and derivation of conversion factors for vasopressor-inotrope equivalent dose.

**Symbol**	**Drug**	**(Units)**	
*N*	Norepinephrine	*mcg*	
*E*	Epinephrine	*mcg*	
*P*	Phenylephrine	*mcg*	
*M*	Milrinone	*mcg*	
*V*	Vasopressin	*units*	
**Conversion Factor**	**Value**	**Derivation of Value (Units)**	**Reference**
	1	$1\left(\frac{Norepinephrine\;Equivalent\;\frac{mcg}{\min}}{Norepinephrine\;\frac{mcg}{\min}}\right)$	(29–31)
*k*_2_	1	$1\left(\frac{Norepinephrine\;Equivalent\;\frac{mcg}{\min}}{Epinephrine\;\frac{mcg}{\min}}\right)$	(29–31)
*k*_3_	0.1	$\frac{1}{10}\left(\frac{Norepinephrine\;Equivalent\;\frac{mcg}{\min}}{Phenylephrine\;\frac{mcg}{\min}}\right)$	(29–31)
*k*_4_	0.5	$\frac{1}{2}\left(\frac{Norepinephrine\;Equivalent\;\frac{mcg}{\min}}{Milrinone\;\frac{mcg}{\min}}\right)$	
*k*_5_	500	$8.33\left(\frac{Norepinephrine\;Equivalent\;\frac{mcg}{\min}}{Vasopressin\;\frac{units}{hr}}\right)\times 60\left(\frac{\min}{hr}\right)$	(29, 31)

### Postoperative Delirium

Delirium for the PATHFINDER study was measured daily until discharge with the Confusion Assessment Method (CAM) (32) in a manner identical to the historical cohort, as described previously (26). The CAM is a diagnostic algorithm for identifying delirium as comprised of acute and fluctuating course, inattention, and at least one of disorganized thinking or altered level of consciousness. Nonverbal (intubated) patients were administered the CAM-ICU (33) which was designed to mimic standard CAM. Assessments were administered by study team members trained in the use of CAM and identification of cognitive impairment.

## Results

### Feasibility, Safety, and Efficacy

All 18 patients in the MMGA cohort were successfully anesthetized using the PATHFINDER MMGA protocol. No adverse effects or complications were noticed due to the anesthetic or antinociceptive regimen. One of the 18 patients experienced complications during recovery unrelated to anesthesia resulting in an extended in-hospital stay and ventilation.

### Hemodynamic Stability and Vasopressor Usage

Intraoperative hemodynamic stability and vasopressor total dose was similar between MMGA and historical cohorts, as quantified by hypotensive AUC (median, [IQR] min'mmHg; MMGA 921.0, [793.1, 1,287.2]; historical 995.1, [653.7, 1,292.7]; *P* = 0.72), CV (median, [IQR]; MMGA 0.32, [0.28, 0.36]; historical 0.34, [0.31, 0.38]; *P* = 0.14), and norepinephrine equivalent dose (median, [IQR] mcg; MMGA 734.5, [526.1, 856.1]; historical 704.8, [336.4 1,093.5], *P* = 0.94). All comparisons were made via two-tailed Mann-Whitney U test, and we note the low statistical power of the small sample size relative to the large spread of values for these observations, as shown in Figure 3.

### Postoperative Delirium and Intraoperative EEG Suppression Events

**Figure 4:**
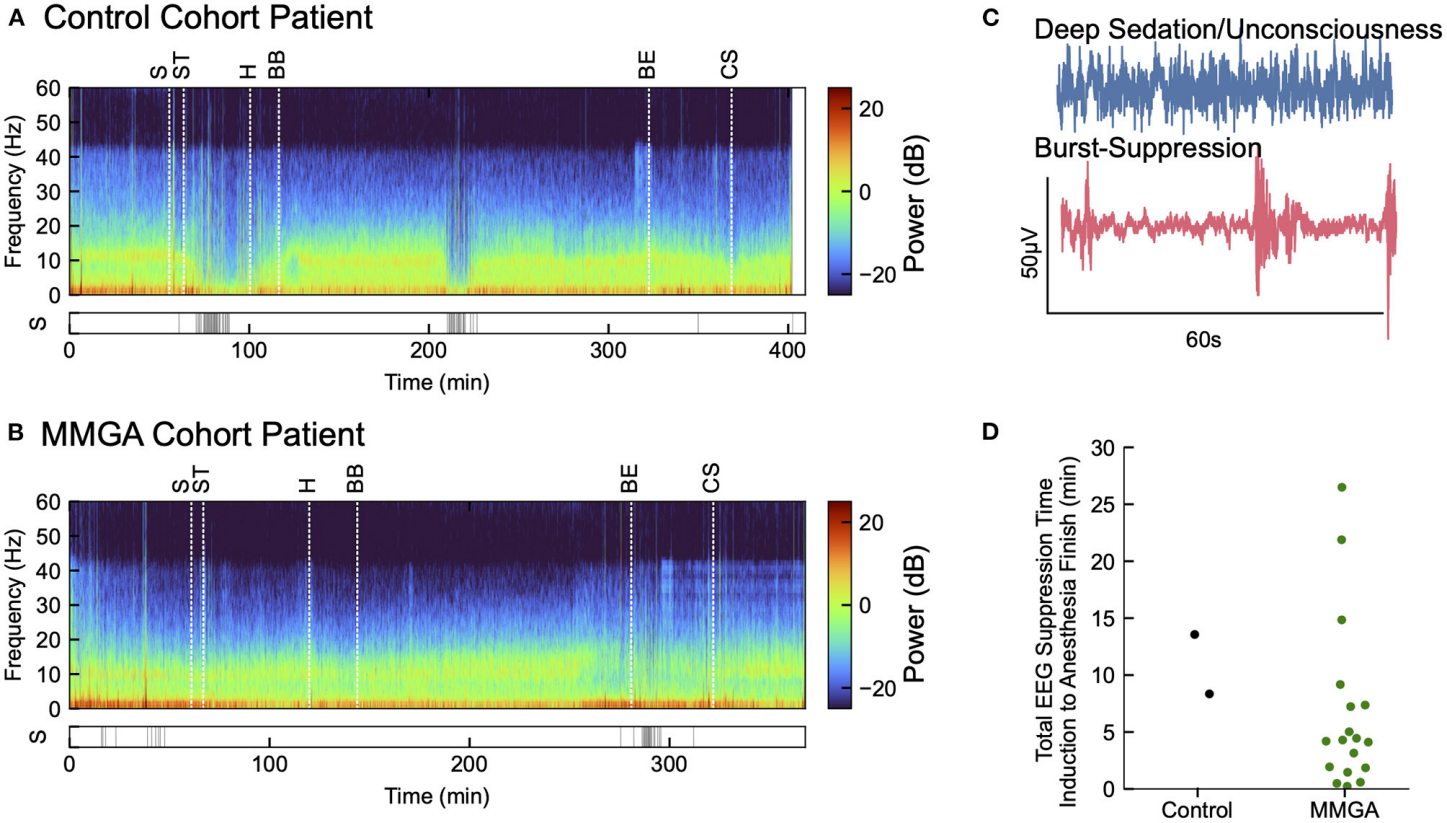
Annotated recordings from one cardiac surgery using MMGA. **(A)** Computed EEG spectrogram for a selected MMGA cohort patient during cardiac surgery with labeled timepoints of surgical incision (S), sternotomy (ST), heparin bolus (H), bypass begin (BB), bypass end (BE), and sternal closure (CS) before ICU transfer. Suppression events are shown below spectrogram. **(B)** EEG spectrogram for a control cohort patient labeled as in **(A)**. **(C)** Selected 60s EEG epochs comparing a waveform during general anesthesia with a waveform during burst suppression, a coma-like deeper state of anesthesia. **(D)** Distribution of the of time spent in suppression from induction to anesthesia end for the MMGA (*n* = 18, median suppression time = 4.24 min, IQR [1.87, 7.33]) and control (*n* = 2, suppression times 13.56 min and 8.35 min) cohorts.

Postoperative delirium was assessed as described in the Methods. Of the historical cohort 17/59 (28.8%) exhibited postoperative delirium. Of the MMGA cohort, 4/18 (22.2%) exhibited postoperative delirium, resulting in no statistically significant difference between the historical and feasibility cohort (*P* = 0.76; Fisher's exact test), however, statistical power was low due to the small sample size used in a feasibility study. One of the four MMGA patients experiencing delirium had an extended hospital stay due to a complication not involving the protocol and was placed on a ventilator for an extended number of days postoperatively, which also interfered with cognitive recovery. We observed suppression EEG patterns infrequently during the MMGA protocol (median suppression time = 4.24 min, IQR [1.87, 7.33]), as shown in Figure 4. The two control patients exhibited suppression patterns for 13.56 and 8.35 min, respectively. Relatively low times in suppression indicate that EEG-guided MMGA obviates the need for excessive sedation, however, this must be tested in comparison with a full control cohort to determine whether it is superior to standard-of-care.

## Discussion

There has been increased interest in multimodal anesthetic protocols as evidence suggests that using more anesthetic agents at lower doses can increase the effectiveness of an anesthetic protocol while minimizing the possible side effects of the agents. General anesthesia for cardiac surgery presents several specific challenges that other types of surgery do not. During particular surgical steps (e.g., aortic cannulation), cardiac surgeons dictate specific hemodynamic goals that obscure the ability of the anesthesiologists to detect potential nociceptive changes. Additionally, during cardiac bypass, hemodynamic signals are lost entirely and cannot be used to adequately titrate anesthetic medications. Therefore, MMGA presents a solution for a general anesthesia strategy that takes advantage of specific central nervous system targets and raw EEG waveform interpretation to potentially optimize antinociception and levels of unconsciousness in cardiac surgical patients.

The aims for this study were to test the feasibility of this protocol and the ability to use EEG to guide dosing for this protocol and to perform exploratory analysis of hemodynamic stability, postoperative delirium, and intraoperative EEG suppression events. Overall, we found this strategy to be feasible in both the cardiac OR and the ICU at our single center. While direct comparisons to historical data were limited due to small sample size and study design, we found that our results for EEG suppression time, intraoperative hemodynamic metrics (CV and AUC), and delirium demonstrated safety and efficacy with no adverse anesthesia events reported during the study. Confirmation of safety enables comparing MMGA to other cardiac surgery approaches under a full clinical trial. We anticipate that as clinicians accrue experience with MMGA control of hemodynamics will improve further. We plan to test our MMGA cardiac anesthesia strategy vs. standard-of-care cardiac anesthesia at our institution, utilizing a larger sample size and randomization to more directly compare EEG suppression time, hemodynamic stability, and postoperative ventilation time (with the potential for safe extubation in the operating room). Additionally, we anticipate that the addition of local antinociceptives during cardiac surgery will reduce intraoperative nociceptive signaling, thus reducing inflammatory responses and pain postoperatively. Thus, we anticipate potential improvement in in-hospital metrics such as length of ICU stay and postoperative analgesic consumption.

Recently, guidelines for Enhanced Recovery After Surgery (ERAS) in cardiac surgery have been established to optimize perioperative outcomes with groups of evidence-based interventions (34). It is our hope that over time, aspects of our MMGA approach for cardiac surgery can be incorporated to further improve the care of cardiac surgical patients.

## Data Availability Statement

The original contributions presented in the study are included in the article/supplementary material, further inquiries can be directed to the corresponding author/s.

## Ethics Statement

The studies involving human participants were reviewed and approved by Beth Israel Lahey Health Institutional Review Board (IRB). The patients/participants provided their written informed consent to participate in this study.

## Author Contributions

AShan obtained data, assisted in data analysis, prepared tables, and wrote major sections of the manuscript. JA performed data analysis, prepared figures, and wrote major sections of the manuscript. SN helped to obtain data, wrote minor sections of the manuscript, and assisted in data analysis. PM helped to obtain data and assisted in data analysis. EW wrote sections of the manuscript. GS developed data analysis techniques. AShar and RB helped to obtain data and perform critical revisions of the manuscript. VR helped to write and performed critical revisions of the manuscript. VS helped to obtain data and develop study protocols along with performing critical revisions of the manuscript. EB developed study protocols and performed EEG consultation along with conceiving, supervising, and editing major sections of this manuscript. BS developed study protocols, obtained data, and performed data analysis along with conceiving, supervising, and writing major sections of this manuscript. All authors contributed to the article and approved the submitted version.

## Funding

This study was supported by NIH/NIA R01AG065554 (to BS), NIH/NIGMS P01GM118269 (to EB), and NIH/NIA F32AG064886 (to JA).

## Conflict of Interest

Masimo Corporation has licensed and paid royalties on intellectual property to Massachusetts General Hospital created by EB. He is also a cofounder of PASCALL, a company developing closed loop physiological control systems for anesthesiology. The remaining authors declare that the research was conducted in the absence of any commercial or financial relationships that could be construed as a potential conflict of interest.

## Publisher's Note

All claims expressed in this article are solely those of the authors and do not necessarily represent those of their affiliated organizations, or those of the publisher, the editors and the reviewers. Any product that may be evaluated in this article, or claim that may be made by its manufacturer, is not guaranteed or endorsed by the publisher.

## Abbreviations

ABP-mean: Mean arterial blood pressure
AUC: Area under the curve for hypotension (ABP-mean < 65 mm Hg)
BIS: Bispectral index-based guidance
CAM: Confusion Assessment Method
CV: Coefficient of variation of blood pressure
CNS: Central nervous system
EEG: Electroencephalography
ERAS: Extended Recovery After Surgery
F: Frontal EEG lead
Fp: Pre-frontal EEG lead
MAP: Mean arterial pressure
MAC: Minimum alveolar concentration
MoCA: Montreal Cognitive Assessment
MMGA: Multimodal general anesthesia
NMDA: N-methyl-D-aspartate receptor
NE: Norepinephrine Equivalent dose of vasopressor
PIFB: Pecto-intercostal fascial regional block
PATHFINDER: Perioperative multimodal general AnesTHesia FocusINg on specific CNS targets in patients undergoing carDiac surgERies
PND: Perioperative neurocognitive disorders
PNS: Peripheral nervous system
α-2: Alpha-2 adrenergic receptor
GABA: γ-amino butyric acid.
